# Oral Health-Related Quality of Life among Refugees: A Questionnaire-Based Study

**DOI:** 10.3390/healthcare12151525

**Published:** 2024-07-31

**Authors:** Katharina Fink, Kais Alkayed, Franz Sebastian Schwindling, Vera Wiesmüller

**Affiliations:** 1 University Hospital for Dental Prosthetics, Medical University of Innsbruck, Anichstraße 35, 6020 Innsbruck, Austria; katharina.fink@i-med.ac.at (K.F.);; 2 Private Dental Practice, 6800 Feldkirch, Austria; 3 University Hospital for Conservative Dentistry and Periodontology, Medical University of Innsbruck, Anichstraße 35, 6020 Innsbruck, Austria

**Keywords:** OHIP, OHRQoL, oral hygiene, refugees

## Abstract

Objectives: The issue of oral health problems among refugees, not only during their journey but also once they arrive at their destination, is a current one. This study aimed to evaluate the oral health-related quality of life (OHRQoL) of refugees, with the intention of highlighting a potential need for improved education and easier access to dental care. Materials and methods: A questionnaire based on the Oral Health Impact Profile (OHIP-14) was distributed via hyperlink on social media, targeting individuals with a refugee background to collect data on their OHRQoL. Results: A total of 401 participants completed the questionnaire. The median OHIP score for all participants was 8 (IQR 2–17) out of a maximum of 56, with 0 representing the best possible OHRQoL. The median OHIP score was 6 (IQR 2–16) for male and 8 (IQR 2–18) for female participants. Conclusion: A comparison of the OHIP score from this study with reference values from non-refugee populations shows an elevated score and, thus, a lower oral quality of life. Clinical relevance: The findings of this study indicate a clear need for enhanced access to dental care for refugees, coupled with an educational initiative aimed at fostering an appreciation of the importance of daily dental hygiene.

## 1. Introduction

Seeking asylum is not a modern phenomenon. “Asylum” originates from the ancient Greek word “asylon”, which means “refuge, fenced territory” [1]. The practice of giving asylum—providing shelter and protection for strangers seeking help—has turned from a mainly religious obligation to an important tool in international state relations [2]. According to figures released by the United Nations High Commissioner for Refugees (UNHCR), 108.4 million people were forcibly displaced from their homes by the end of 2022 [3], with most of these refugees (6.5 million) coming from Syria [4]. One of the most common reasons for forced migration is war, which promotes the dispersal of diseases due to the destruction of medical infrastructure, the migration of medical staff abroad, and the interruption of drug supply chains [5].

The general and mental health of refugees has been well studied [6,7,8], but their oral health has received less attention. Oral health is affected by the availability of dental services as well as differences in personal behavior, such as smoking, alcohol consumption, dietary habits, and oral hygiene [9]. In Syrian refugees, reduced oral hygiene and higher incidences of caries, periodontal disease, and odontogenic infections have been reported [10]. This has largely been attributed to the low prioritization of dental treatment over other health issues in refugees [11]. Refugees are often forced to spend long periods of time in refugee camps with poor food supply, sanitation, and medical treatment. Although most refugees undergo a general health screening upon arrival at these camps, this usually does not include a dental inspection [12,13,14]. Continuous and adequate access to basic oral hygiene products remains a challenge in these refugee camps [14]. Furthermore, a considerable number of refugees are unable to access dental care in their country of asylum due to a lack of insurance coverage or financial resources. This can present a significant barrier to receiving oral rehabilitation services [15,16].

Oral diseases, dental pain, and tooth loss have an important influence on daily life [17]. They can be directly linked to general health, as good oral health can exert a beneficial effect on the prevention of chronic diseases such as diabetes and cardiovascular diseases. Furthermore, dental pain or loss of teeth can result in psychological distress, reduced social interactions, depression, decreased performance in daily activities and reduced nutrition [9,18]. As well as the physical, there are also psychological aspects to the aesthetic appearance of teeth: stained, misaligned, or missing teeth can affect a person’s self-esteem and self-confidence, leading to a lack of confidence in social interactions. Aesthetic concerns with teeth can also affect professional success. Research suggests that people with healthy and attractive smiles are perceived as more confident, competent, and successful in the workplace [11].

These effects can be evaluated by measuring the oral health-related quality of life (OHRQoL). It should be noted that oral health-related quality of life is not equivalent to patient satisfaction. Rather, it captures the impact of various oral health conditions on functional outcomes and psychosocial well-being. Several questionnaires have been developed to measure OHRQoL, including the Oral Health Impact Profile (OHIP) [17]. The OHIP is based on the OHRQoL model of Locker (1988) and includes 49 questions [19] with Likert scale scores from 0 to 4 assessing functional limitation, pain, psychological discomfort, and disrupted social ability.

The aim of this study was to evaluate the OHRQoL and oral hygiene habits of refugees using the 14-item OHIP questionnaire (OHIP-14) to serve as a reminder to host countries of the importance of ensuring accessible healthcare and, in particular, dental care for refugees. We hypothesized that individuals with a refugee background would have a higher OHIP score than non-refugees in contrastive studies, indicating a lower OHRQoL.

## 2. Material and Methods

### 2.1. Study Design and Ethics Statement

OHRQoL was investigated using an online questionnaire based on the OHIP-14. This study was approved by the Ethics Committee of the Medical University of Innsbruck, Austria (1425/2021) and was conducted in accordance with the 1964 Helsinki Declaration and its later amendments. The questionnaire was presented only to those individuals who had signed the declaration of consent and the data privacy statement approved by the Medical University of Innsbruck by ticking the appropriate box. Consequently, the data collected were limited to participants who had actively consented to this study. As forced movements also affect many young people, the Ethics Committee gave us permission to include individuals from the age of 14 years.

### 2.2. Data Collection

Since this was a pilot study, we aimed to maximize the number of possible participants, and no sample size was calculated. To gather data on the OHRQoL of refugees, the OHIP-14 was transformed into a web-based questionnaire including questions on oral health habits. The web-based questionnaire was developed using Research Electronic Data Capture (REDCap) and was distributed via hyperlink to relevant groups (such as “Die weißen Hände in Tirol” and “freie Syrer Koordination”) on social networks and messenger services. Data were collected from 17 May until 30 June 2022 by V.W. and K.A.

All refugees aged 14 and above, residing in Austria for a maximum of ten years, were invited to participate in the survey and could complete the questionnaire in either Arabic or German. Potential participants were informed of the background, aim, and data processing before inclusion, and the questionnaire was only made available to those individuals who signed the declaration of consent and the data privacy statement approved by the Medical University of Innsbruck.

### 2.3. Questionnaire Design

The questionnaire was divided into two parts. In the first part, data were collected on demographics and oral cleaning, smoking, eating, and drinking habits. In the second part, the OHIP-14 was used to examine how oral health affected life quality in seven domains of personal limitation: functional limitation, physical pain, psychological discomfort, physical disability, psychological disability, social disability, and speech limitation (disability). The questionnaire was provided as Supplementary Material. The OHIP 14 is a condensed iteration of the original, globally utilized, multi-controlled, and highly time-efficient version. This questionnaire generated OHIP scores ranging from 0 to 56. Participants were asked to rate their oral health on a scale of 0–4, where 0 = “never”, 1 = “hardly”, 2 = “every now and then”, 3 = “often”, and 4 = “really often”, with a score of 0 indicating the best possible oral quality of life and 56 indicating the worst possible oral quality of life. The questionnaire was distributed online in both German [20] and Arabic, which allowed us to gather a large cohort of refugees. 

### 2.4. Statistical Analysis

Statistical analysis was conducted using IBM SPSS Statistics Version 28 (IBM Corp. Armonk, NY, USA, 2021). Qualitative data are presented as relative and absolute frequencies, while quantitative data are presented as medians with interquartile ranges (IQRs). For comparison, on the one hand, quartile-based groups, and on the other hand, the group with optimal OHIP (score = 0) were compared with participants with higher OHIP values (restrictions in oral health-related quality of life). Groups were compared using the Chi^2^, Mann–Whitney U, and Kruskal–Wallis tests and logistic regression analysis, and *p*-values < 0.05 were considered statistically significant.

## 3. Results

Five hundred and forty-two individuals participated in this study; seventy-one were excluded because they did not consent to participate. Participants were allowed to skip questions, and any skipped answers were considered not applicable. The median (IQR) age was 23 (20–29) years (min 15–max 75). A total of 66.2% of the participants identified as female and 32.1% as male, and most (83.9%) originated from the Middle East, with 77.1% emigrating from Syria (see Table 1).

A total of 401 of the 471 participants completed the 14-item Oral Health Impact Profile (OHIP-14) in the second part of the questionnaire, 259 of whom were female. The data were not normally distributed according to the Shapiro–Wilk test, and thus, they were described using medians and IQRs.

The median of the OHIP scores in all participants was 8 (2–17) out of a maximum of 56, with 0 representing the best possible OHRQoL. The median OHIP score was 6 (2–16) in males and 8 (2–18) in females. Table 2 shows the OHIP scores and percentage distribution of OHIP-14 responses in the seven domains of personal limitation. The most affected domain was physical pain, with a median score of 2 (0–4). The least affected domain was functional limitation, with a median score of 0 (0–1).

When asked about their current dental health, 15.7% of participants reported it as very good, 47.1% as good, and 18.7% as satisfactory, whereas 12.5% reported it as rather poor, 3.2% as poor, and 2.1% as very poor. We compared this self-perceived oral health status with OHIP scores and found that 76.8% of participants with OHIP scores of 0–2, 73.8% of participants with OHIP scores of 3–8, and 62.2% of participants with OHIP scores of 9–17 reported very good or good self-perceived oral health. A total of 41.1% of participants with OHIP scores > 18 still reported their oral health status as very good or good. A comparison of self-perceived oral health status as indicated by a single question and the results of the OHIP-14 revealed a statistically highly significant difference (*p* < 0.001).

With regard to dental hygiene, 59.9% of respondents reported attending their dentist annually for professional tooth cleaning (see Table 3). There was no statistically significant difference between the OHIP scores of those who had an annual professional tooth cleaning and those who did not (*p* = 0.239). We also found that 51.8% of participants brushed their teeth at least twice a day, either manually or with an electronic toothbrush. OHIP scores were significantly lower in individuals who brushed their teeth at least twice daily (median, 6; range 2–15.75) than in those who did not (median, 9; range, 3–19.75) (*p* = 0.035). A more pronounced statistically significant difference with regard to brushing behavior is demonstrated by a logarithmic regression analysis, which compares individuals with an OHIP score of 0 (no abnormalities) with individuals with a score of 1–56 (limitations in oral health-related quality of life) (*p* < 0.001), indicating a highly statistically significant difference in brushing either manually or with a powered toothbrush. Furthermore, 30.8% of participants reported using interdental cleaning products at least once daily, including floss, floss with stiff ends, interdental brushes, and toothpicks. OHIP scores did not differ between those who performed daily interdental cleaning and those who did not (*p* = 0.952).

Due to the wide range of age of participants, the correlation of age and the OHIP score was assessed. There was no statistical correlation between age and OHIP results (*p* = 0.206 quartile-based; *p* = 0.068 for OHIP score 0/OHIP score 1–56).

## 4. Discussion

In this study, we investigated the self-perceived status of oral hygiene and OHRQoL among refugees using an online questionnaire.

The OHIP-14 score in this study (median 8; interquartile range 2–17) was higher than that published in a German study conducted in 2004 to establish OHIP-14 standards. That study differentiated between participants without dentures (mean score, 0; 90th percentile, 11), with partial dentures (mean score, 4; 90th percentile, 17), and with full dentures (mean score, 6; 90th percentile, 25) [21]. In 2021, a study in the *Journal of Oral Rehabilitation* found that the minimal important difference (MID) for a clinically relevant OHIP-14 score is two points. Based on this, the higher OHIP scores we observed are clinically significant [22]. The findings of this study are in alignment with those of two previous studies (2018, 2021) which indicate a higher prevalence of oral health issues among refugees than among the average population of the host country [16,23].

**Figure 1:**
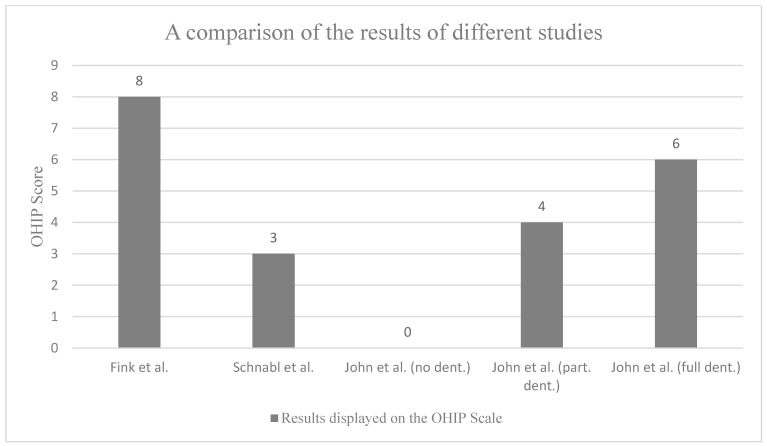
A comparison of the results obtained with the 14-item version of the Oral Health Impact Profile. The scores range from 0 to 56, with 0 indicating the best possible oral quality of life and 56 indicating the worst possible oral quality of life. The findings of this study (Fink et al.) indicate a higher OHIP score than that observed in other studies [21,24].

Other studies have also used the OHIP scale to measure OHRQoL (see Figure 1). In 2018, a study by the Medical University of Innsbruck used the OHIP scale and found an OHIP score of 3 (0–6) among dental students. In this collective, male participants had lower scores (median, 2; IQR, 0–4) than females (median, 4; IQR, 1–11) [24]. In 2019, another study compared OHIP scores between native and immigrant populations in Spain. They found significantly lower OHIP scores in native women (median, 6; IQR, 11) than in immigrant women (median, 15.5; IQR, 21), but no differences between native (median, 4; IQR, 9) and immigrant (median, 5; IQR, 8) men. Psychological discomfort was also rated higher by women than by men [25].

A possible reason for these higher OHIP-14 scores and, therefore, worse OHRQoL among refugees could be difficulties accessing the medical system because of language barriers or insurance issues [18,26,27]. In a paper on refugees’ access to the medical system, one refugee described how inefficient their medical treatment was and how poor the communication was with health care providers: “The first time I went to the social office […] the lady in the social office started speaking German so I didn’t understand what she wanted […]. And then I had many other appointments with the doctor and every time he gave me something to […] numb the pain […] But he didn’t fix it” [18]. This problem was emphasized in a recent scoping review, which reported a higher prevalence of caries and periodontal problems in refugees, as well as a very low level of accessibility to dental health services, with 17–72% of refugees having never visited a dentist [28]. These findings are supported by a systematic review published in 2022, which concluded that insufficient food intake and poor dietary diversity are common among refugees and can lead to deficiency symptoms, chronic diseases, and poor oral health [29].

Another reason for lower OHRQoL among refugees could be different cultural attitudes to oral health and practices. Indeed, different OHIP-14 scores have been reported among different populations. A study using the Persian version of the OHIP-14 found a median OHIP score of 12 in 400 individuals (50% male; mean age, 35.8 ± 12.8 years) [26]. Another study conducted in Saudi Arabia among college students examined OHIP-14 scores and found the highest scores for physical pain (mean, 4.14), followed by psychological discomfort (4.07) and psychological disability (3.73) [30]. In another study, Arabic-speaking people who had recently moved to the US had poorer oral health and practices than natives did [25].

Regarding oral health practices, only 52.1% of participants in our study claimed to brush their teeth at least twice a day, and only 59.9% visited their dentist for regular checkups. This exceeds the oral health behavior described in Saudi Arabian college students, where only 39.9% of the sample regularly visited their dentist and only 40.6% brushed their teeth twice a day [30]. Nevertheless, apart from cultural customs, OHIP scores were significantly lower, and therefore the oral quality of life was better, in individuals who brushed their teeth at least twice daily (median, 6; range 2–15.75) than in those who did not (median, 9; range, 3–19.75) (*p* = 0.035).

A German study compared the oral health behavior of elderly migrants and non-migrants. A total of 82.4% of the non-migrant population and 55.7% of the migrant population reported to have a dentist that they visit regularly. The greatest barrier to accessing dental care for migrants was identified as cost, with 31.1% of respondents citing this as a reason. In contrast, only 7.8% of non-migrant respondents indicated that cost was a barrier to accessing dental care [31].

We found that most of our participants believed their oral health to be excellent or good in answer to a single question, even those with OHIP scores > 18. The statistically significant difference between perception for a single question and a questionnaire such as the OHIP-14 (*p* < 0.001) demonstrates that a detailed questionnaire is necessary to obtain realistic self-perception of oral health status.

There are some limitations to this study. First, we did not distinguish between participants with and without dental prostheses, although this has been found to have a significant impact on OHIP scores [32]. We excluded the question on dental prostheses from our questionnaire because a clinical examination was not possible, and we found that many patients did not understand dental terminology and could not reliably state the type of prosthesis they were wearing. The median age of our cohort was 23 (20–29) years, suggesting that most participants were not likely to be wearing dental prostheses. A second limitation to this study is that the gender distribution was uneven, with almost two-thirds of the questionnaires being completed by women. It has been discovered that a significant number of the targeted social media groups were predominantly accessed by women, which could account for the observed increase in the proportion of women in this study. Two recent studies have shown that women tend to use social media more than men. In particular, Facebook, Instagram, and WhatsApp appear to be used more by women than men, which may be another reason why more women were attracted to completing the form due to its distribution through Facebook and WhatsApp [33,34]. A third limitation of this study was that participants were allowed to skip questions, which made it challenging to accurately compare the results. To address this, we only analyzed those questionnaires that were completed in their entirety.

## 5. Conclusions

In conclusion, the evidence presented indicates that individuals with a refugee background have a lower oral health-related quality of life, as they appear to have a higher OHIP score than individuals with no refugee background in comparison studies. Our findings highlight the need for improved access to dental care for refugees and for education on the importance of daily dental hygiene among these individuals, as our results indicate that the frequency of dental cleaning, performed two times per day, is associated with a better oral health-related quality of life. Our results represent a call to action for host countries to address these issues.

## Figures and Tables

**Table 1:** Distribution of age, sex, and country of origin.

Age (years)	Median (IQR)	23 (20–29)
	Minimum	15
	Maximum	75
Sex	Male	32.1%
	Female	66.2%
	Not applicable	1.7%
Country of origin	North Africa (Libya, Egypt, Morocco, Tunisia, Algeria, Sudan)	4%
	East Africa (South Sudan, Tanzania)	0.4%
	Arabian Peninsula (Yemen, Qatar, Oman, Saudi Arabia, United Arab Emirates, Kuwait)	3.1%
	Middle East (Iraq, Jordan, Lebanon, Syria, Israel)	83.9%
	Not applicable	0.8%

**Table 2:** Results of the 14-item Oral Health Impact Profile (OHIP-14) for all participants, divided into seven domains of personal limitation.

Domains	OHIP Scores Median (IQR)	OHIP Questions	Never (%)	Hardly (%)	Now and Then (%)	Often (%)	Very Often (%)
Total OHIP-14	8 (2–17)						
Domain 1: Functional limitation	0 (0–1)	Had difficulties pronouncing words	78.6	10.2	6.0	3.5	1.7
		Felt limitation in sense of taste	84.3	10.2	3.5	0.7	1.2
Domain 2: Physical pain	2 (0–4)	Felt pain in oral region	34.4	21.4	28.4	8.0	7.7
		Felt uncomfortable eating certain foods	53.4	14.7	20.0	6.0	6.0
Domain 3: Psychological discomfort	1 (0–3)	Felt insecure related to your teeth or dentures	46.1	16.0	23.7	6.7	7.5
		Felt tense	61.3	15.0	13.2	5.2	5.2
Domain 4: Physical disability	1 (0–3)	Felt diet has been unsatisfactory	58.6	8.0	17.0	7.7	8.7
		Had to interrupt meals	59.6	17.5	15.2	4.5	3.2
Domain 5: Psychological disability	1 (0–3)	Had difficulties relaxing	56.6	13.0	16.5	7.5	6.5
		Felt embarrassed	58.4	12.7	13.7	8.0	7.2
Domain 6: Social disability	0 (0–3)	Felt irritated by others	63.1	11.7	13.0	5.5	6.7
		Had difficulty with daily routine	66.3	14.2	12.0	4.2	3.2
Domain 7: Handicap	0 (0–2)	Felt life in general less satisfying	61.8	11.0	15.2	4.5	7.5
		Been totally unable to function	78.3	10.5	6.2	2.5	2.5

**Table 3:** Oral health habits of the total cohort and the quartile-dependent summary of the OHIP scores in groups. Analysis only possible for cases that provided information on the respective oral hygiene product.

	Overall	OHIP 0–2	OHIP 3–8	OHIP 9–17	OHIP > 18	*p*
Professional cleaning 1×/year	59.9%	63.0%	62.3%	62.6%	53.1%	0.239
Brushing at least 2×/day	52.1%	55.6% *	58.5% *	43.3% *	44.8% *	0.035 *
Manual toothbrush at least 2×/day	44.9%	44.5%	50.0%	36.3%	43.8%	0.258
Electric toothbrush at least 2×/day	11.0%	13.0%	10.3%	9.9%	5.2%	0.274
Interdental cleaning at least 1×/day	33.8%	28.2%	38.2%	38.1%	27.5%	0.952
Interdental brushes at least 1×/day	19.8%	16.7%	23.6%	25.3%	14.0%	0.399
Tooth floss at least 1×/day	16.3%	15.7%	17.9%	18.7%	11.4%	0.254

* statistical significance.

## Data Availability

All collected data were provided in this publication.

## Supplementary Materials

The following supporting information can be downloaded at: https://www.mdpi.com/article/10.3390/healthcare12151525/s1, Questionnaire.
