# Supplementary material for: Oral Health-Related Quality of Life among Refugees: A Questionnaire-Based Study
Source: Healthcare (Basel). 2024 Jul 31;12(15):1525. doi: 10.3390/healthcare12151525 (PMC11311352; doi:10.3390/healthcare12151525)
Supplement: Supplementary file 1 [file healthcare-12-01525-s001.zip › healthcare-3056435-supplementary.pdf]

Questionnaire (German version, translated into English by Dr. Fink)

1. Gender:

a. Male
b. Female
c. Diverse
d. Not specified

2. Age:

Dropdown (years 14 - 99)

3. Country of origin (drop down):

a. Egypt
b. Algeria
c. Ethiopia
d. Bahrain
e. Djibouti
f. Eritrea
g. Gambia
h. Iraq
i. Iran
j. Israel
k. Yemen
l. Jordan
m. Qatar
n. Comoros
o. Kuwait
p. Lebanon
q. Libya
r. Mali
s. Morocco
t. Mauritania
u. Niger
v. Nigeria
w. Oman
x. Palestine
y. Saudi Arabia
z. Senegal
aa. Somalia
bb. Sudan
cc. South Sudan
dd. Syria
ee. Tanzania

ff. Chad
gg. Tunisia
hh. Turkey
ii. United Arab Emirates
jj. Cyprus
kk. Not specified

<u>4. Professional status:</u>

a. employed
b. self-employed
c. asylum procedure not completed
d. (compulsory) school
e. studies (university/university of applied sciences)
f. other training (e.g. apprenticeship)
g. housewife / housekeeper
h. unemployed
i. not specified

<u>5. Highest completed school education:</u>

a. compulsory school leaving certificate
b. high school diploma
c. apprenticeship certificate
d. university/university of applied sciences degree
e. not specified

<u>6. Highest level of education completed by your parents:</u>

a. compulsory school leaving certificate
b. high school diploma
c. apprenticeship certificate
d. university/university of applied sciences degree
e. no answer

<u>7. How do you feel about the health of your teeth?</u>

a. Very good
b. Good
c. Satisfactory
d. Rather poor
e. Poor
f. Very bad

8. How often do you use the following oral and dental care products?

	Never	Once a month	Once a week	2-3 times a week	Once a day	Twice a day	More than twice a day	I do not know
Manual toothbrush								
Electric toothbrush								
Interdental brush								
Dental floss								
Dental floss with stiffened ends (Superfloss)								
Mouthwash								
Toothpicks / Miswak								
Oral irrigator								
Miscellaneous								

9. Have you ever been shown how to brush your teeth properly by your dentist or prophylaxis assistant?

a. Yes
b. No

10. Do you have your teeth professionally cleaned regularly (once a year or more)?

a. yes
b. no

11. How often do you consume sweets or sugary drinks (soft drinks/fruit juices)?

a. Several times a day
b. Once a day
c. Several times a week
d. Rarely
e. Never

12. How often do you drink tea or coffee with sugar?

a. Several times a day
b. Once a day
c. Several times a week
d. Rarely
e. Never

13. How often do you smoke?

a. > 20 cigarettes a day
b. 10-20 cigarettes a day
c. < 10 a day
d. Rarely
e. Never

14. OHIP-14 Questionnaire on Oral Health Quality of Life

	Very often	often	From time to time	hardly	never

In the past month, have you had any problems with your teeth, mouth or dentures...					
Difficulty pronouncing certain words	4	3	2	1	0
the feeling that your sense of taste was impaired?	4	3	2	1	0
the impression that your life was generally less satisfying?	4	3	2	1	0
Difficulty relaxing?	4	3	2	1	0
**In the past month, due to problems with your teeth, mouth or dentures, did it happen…**					
that you felt tense?	4	3	2	1	0
that you had to interrupt your meals?	4	3	2	1	0
that you felt uncomfortable eating certain foods?	4	3	2	1	0
that you were rather irritable towards other people?	4	3	2	1	0
that you found it difficult to carry out your everyday activities?	4	3	2	1	0
that you were completely unable to do anything?	4	3	2	1	0
that you felt a little embarrassed?	4	3	2	1	0
that your diet was unsatisfactory?	4	3	2	1	0
**In the past month, have you had...**					
- pain in the mouth?	4	3	2	1	0
- a feeling of insecurity in connection with your teeth, your mouth or your dentures?	4	3	2	1	0

15 Please rate the following statements on the subject of oral health as "Agree" or "Disagree". If you cannot answer the question, please mark the answer that you think is most correct:

1. Sugar consumption is the main cause of tooth decay.

a. Agree

b. Do NOT agree

2. Bleeding from the gums means that the gums are diseased.

a. Agree

b. Do NOT agree

3. you can prevent gingivitis and tooth loss by brushing your teeth.

a. Agree

b. Do NOT agree

4. oral hygiene has an influence on the quality of life.

a. Agree

b. Do NOT agree
